# The Emergence of Predators in Early Life: There was No Garden of Eden

**DOI:** 10.1371/journal.pone.0005507

**Published:** 2009-06-03

**Authors:** Silvester de Nooijer, Barbara R. Holland, David Penny

**Affiliations:** Allan Wilson Center for Molecular Ecology and Evolution, Massey University, Palmerston North, New Zealand; Max Planck Institute for Evolutionary Anthropology, Germany

## Abstract

**Background:**

Eukaryote cells are suggested to arise somewhere between 0.85∼2.7 billion years ago. However, in the present world of unicellular organisms, cells that derive their food and metabolic energy from larger cells engulfing smaller cells (phagocytosis) are almost exclusively eukaryotic. Combining these propositions, that eukaryotes were the first phagocytotic predators and that they arose only 0.85∼2.7 billion years ago, leads to an unexpected prediction of a long period (∼1–3 billion years) with no phagocytotes – a veritable Garden of Eden.

**Methodology:**

We test whether such a long period is reasonable by simulating a population of very simple unicellular organisms - given only basic physical, biological and ecological principles. Under a wide range of initial conditions, cellular specialization occurs early in evolution; we find a range of cell types from small specialized primary producers to larger opportunistic or specialized predators.

**Conclusions:**

Both strategies, specialized smaller cells and phagocytotic larger cells are apparently fundamental biological strategies that are expected to arise early in cellular evolution. Such early predators could have been ‘prokaryotes’, but if the earliest cells on the eukaryote lineage were predators then this explains most of their characteristic features.

## Introduction

The origin of the eukaryote cell is often suggested to occur anywhere from 850 to 2700 Mya [1], [2]. However, in the present world of unicellular organisms, cells that derive their food and energy from engulfing smaller cells (phagocytotic predation, or simply predation in our sense) are almost exclusively eukaryotic. It is of course well-known that there are bacteria, for example, that attack and consume others [3] but our interest here is in phagocytotic predators – larger cells that engulf small cells. Combining the propositions that, eukaryotes were the first predators that engulfed smaller cells, and that they arose only 0.85∼2.7 billion years ago, leads to an unexpected prediction of a long period (∼1–3 billion years) without predators. Such a lack of predators for somewhere between 1–3 billion years is a Garden of Eden (or Shangri-La, [4]), and is reminiscent of “The wolf and the lamb shall feed together, and the lion shall eat straw like the bullock” (Isa 65:25).

There are many theories for eukaryote origins (reviewed in [5]) but some earlier ones ignored basic life history and ecological principles to the extent that they had intracellular parasites (such as Microsporidia) being ancient lineages that existed long before their multicellular hosts! Recent reviews do stress the need for considering ecological and life history traits [5], [6] and there are many reasons to be suspicious [see discussion in 7] of overly simplistic hypotheses for the origin of features such as the eukaryote nucleus, with its associated complex splicing machinery [8], large numbers of introns and exons [9], [10], and many protein families unique to eukaryotes [11], [12]. Rather than revisit those issues, we simply examine the prediction that there was a long period, early in life, with no predators. Is such a period expected from first principles?

Although the above scenario is the starting point for the present investigation, it is much better to put this eukaryote origin example aside, and concentrate on the underlying biological principles. To a biologist it is suspicious that there would be any period of time, however remote, that normal biological and ecological principles did not apply. This conclusion is reinforced by the knowledge that many ecological principles are fundamental and can, in principle, be derived directly from thermodynamics [13]. Simulation is a standard approach in evolution to understand the fundamental principles behind empirical observations, and has the advantage that a very wide range of parameters can be studied [14]. Important examples include properties of quasi-species and hypercycles [15], increased fidelity of replication [16], optimal numbers of nucleotides [17], scaling laws [18], origin of cooperation [19], [20], properties of genetic distances [21], public goods [22], reciprocal altruism [23], promotion of biodiversity [24], and the emergence of species with similar sizes [25]. Thus the simulation approach is powerful in studying the emergence of biological features from basic principles, and here we study what properties might emerge under a wide range of starting conditions.

In order to test whether fundamental principles are expected to lead to specialization, we created a simulation of simple basic cells that adhere to physical and biological principles. The properties upon which we have based our model are all standard within biology, and include the following:

(*conservation of energy and matter*); energy and food is introduced to the system only through food assimilated by the unicells, after which it can flow through the food chain. At every level of the food chain some energy is lost as waste products from assimilation inefficiency, metabolism, growth and cell death.
*(surface to volume ratio)*; smaller primary producers are more efficient in terms of nutrient and gas exchange because of their larger surface area to volume ratio [4].
(*power law of metabolism*); metabolism scales with weight to the power of ¾ [26].
(*inheritance*); except for the initial population, all unicells are progeny of other unicells with inherited properties; the property of cell size allows mutations.
(*predation advantage*); predation provides a larger energy gain per unit feeding time than does primary production; this is balanced against fewer feeding opportunities.
(*size asymmetry*); if there is engulfment the ‘predator’ needs to be larger than the ‘prey’ [27]. We require that the ratio of predator/prey size exceeds a threshold for predation to occur. Conversely, this means that larger size also provides protection against predation [28].
(*positional information*); the simulation preserves positional information because opportunities for predators or parasites depend on their geographical location with respect to their prey [15].

Additional details are in Materials and Methods. Because we are focusing on the principles the simulations do not explicitly include pathogens (such as viruses) or saprophytes (that break down dead material). However, these are covered implicitly in that, for example, cells may die at random from such things as viral infection (random death, see Materials and Methods) and waste products (including dead unicells) are removed from the simulation. We test whether the above principles are sufficient to give rise to cellular specialization, including phagocytosis. Our unicells (which we call weebeasties) are kept as simple as possible while adhering to the above principles.

## Results

**Figure 1:**
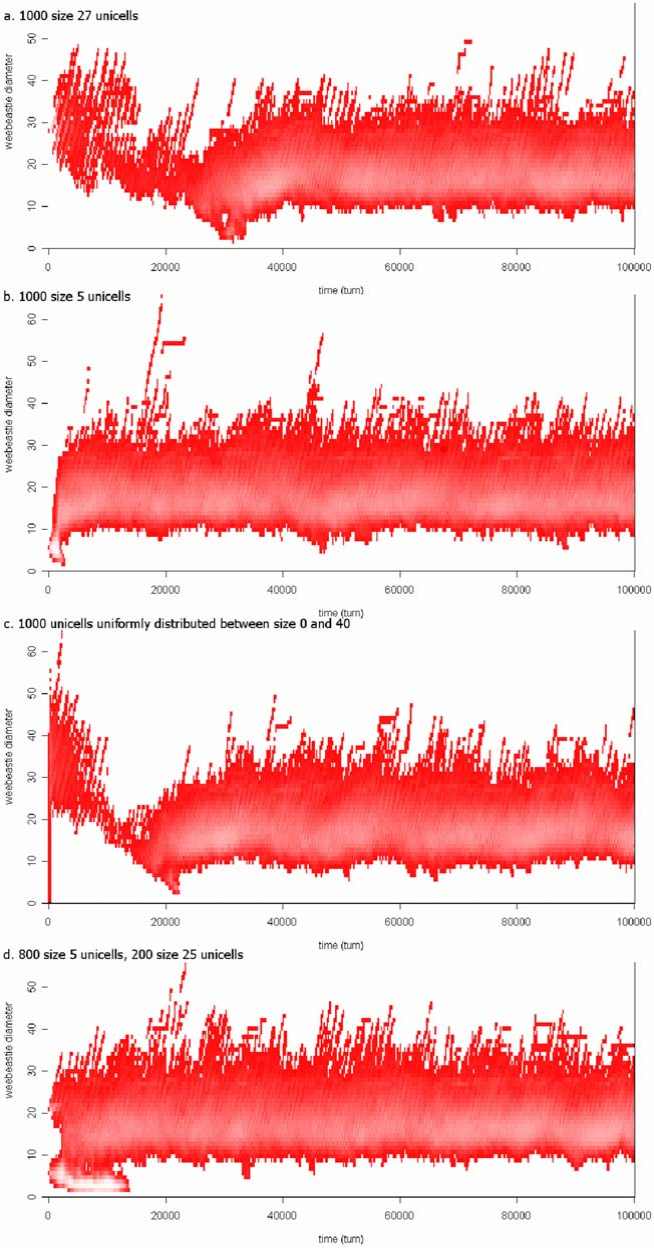
A stable size distribution is reached from different starting points. The vertical-axis shows unicell diameter, the x-axis is time expressed in simulation turns. The intensity of the color, ranging from dark to light red on a logarithmic scale, shows the frequencies of unicells in each size class. Although the beginning situations (1a–1d) are very different, all 4 simulations converge to similar population dynamics. In 1a and 1b all cells start at the same size but are larger (1a) or smaller (1b) than the final average size. In 1c and 1d the sizes of the initial unicells are selected from either a uniform (1c) or bimodal (1d) distribution. Additional information on conditions and parameter values are described in Supplementary Information.

The first conclusion is that, virtually independently of the starting conditions of the simulation, there is specialization into smaller primary producers and larger predators that engulf smaller cells; that is, the simulations converge to the same state from a wide range of initial conditions (Figure 1). These four panels show a range of starting sizes of unicells, varying from all cells being of the same size but either larger (1a) or smaller (1b) than the final average size. Similarly, the sizes of the initial unicells can be selected from a distribution, either uniform (1c) or bimodal (1d). After an initial period we find that there is no net change in the size distribution of unicells; the results fluctuate around a dynamic equilibrium (from a statistical viewpoint, the distribution is ergodic). This distribution is one of two main stable states observed in the simulation: the other being extinction when the food supply is very low.

Figure 1 shows the total distribution and thus averages out any local population dynamics. However, because of the limited spatial size in the experiments, predator-prey cycles in subpopulations can be observed in the banding pattern of lighter or darker striations. There are phases with an increased maximum size and abundance of the top predators. However, the largest predators can run out of food and die, allowing the small unicells to increase in number again. Thus the banding patterns (striations) in Figure 1 are highly informative about the underlying processes.

**Figure 2:**
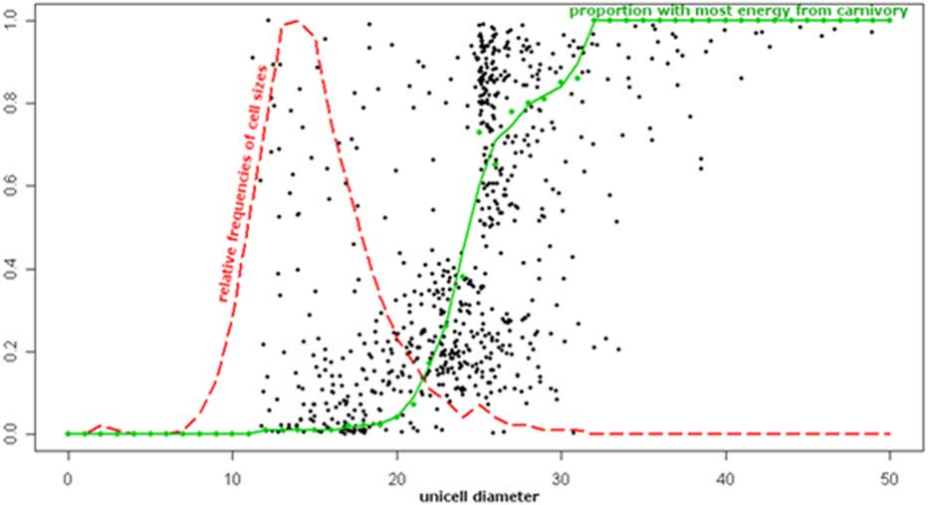
Proportion of energy derived from predation. The dashed red line shows the relative proportion of unicells in each size class; the large majority of unicells are in the 10–20 range. Black dots are the proportion of energy derived from predation for the ∼4% of individual unicells that have obtained some food from predation. (Not shown separately are the vast majority of unicells, ∼96%, that have derived all their energy from primary production.) The green solid line is the fraction of unicells in each size class that have derived the majority of their energy from predation. Only the rare cells larger than about 24 units obtain most of their energy from predation. Parameters and conditions are described in Supplementary Information, table 2.

We next consider a single population in more detail. Figure 2 is a snapshot of a simulation at one point in time, and shows that size is the major factor with regard to the ecological niche of a unicell. The dashed line shows the relative proportion of unicells in each size class. In this simulation, around 96% of unicells have derived all their energy/food from primary production and these are not shown individually on the graph. However, each unicell that has obtained some food by predation is plotted by its volume (x-axis) and the fraction of its energy derived from carnivory (y-axis).The results emphasize both that most unicells are small, and that these small cells are almost exclusively primary producers. In contrast, the solid line shows the proportion of unicells in each size class that have derived the majority of their energy and food from predation.

The vast majority of unicells are small; being small means a faster replication time (food uptake-rate scales with surface area whereas the amount of growth required prior to division scales with volume). In this simulation, primary producers of diameter 11 replicate on average every 287 iterations, while those of size 18 replicate every 530 and those of size 24 replicate every 666 iterations, providing a clear advantage to smaller unicells in replication rate. The increase in generation time varies with simulation conditions but is not generally linear with cell diameter. Under the settings illustrated, replication time tends to increase exponentially for the smaller (primary producer) cells and then to level off around size 20. In the present simulation (Figure 2), most unicells greater than size 24 are carnivores. Generation time again rises steeply for the largest unicells. However, by that point the results become somewhat noisy because of the small number of very large cells in the simulation.

Again, in this simulation (Figure 2), only 4% of all unicells have consumed another unicell and only half of these (2% overall) derive the majority of their energy from predation. The distribution of predation versus size shows subpopulations. A cluster of opportunistic predators resides between roughly size 20 and 25, while a cluster of specialist predators resides between size 25 and 28. Given that in this particular simulation, a cell had to be twice the diameter of a smaller cell in order to consume it, the size range 25–28 is the smallest that gives a fair chance of encountering prey sufficiently small for them to engulf. There are virtually no unicells smaller than size 11 but an abundance of potential prey with size 12–14. As demonstrated by the green line, and although they are a very small proportion of total cells, virtually all unicells beyond size 33 derive the majority of their food from predation. At this size their surface area/volume ratio is small and their rate of food uptake across their surface is not sufficient to offset normal loss through metabolism or ‘random death’ (at these large size the reproduction rate of the unicells is very slow, leading to more opportunities for an infection and/or death).

Figures 1 and 2 include the major results and lead to the conclusion that, in principle, there is a viable niche for predators in our simulations and consequently in any biological system that adheres to the same basic physical and ecological principles. In order to ensure that our results are general, and do not occur just in a very small subset of the parameter space, we tested a wide range of values for the parameters. Over this wide range of values, and after an initial phase, only three stable situations are observed: extinction, a limited range with only primary producers, and a population that includes both primary producers and phagocytotic predators. In addition, a few populations continue to evolve towards larger sizes.

The simplest conditions leading to extinction have a severe restriction of the energy/food supply (compared to their expenditure on metabolism) and results in starvation (see Supplementary Figure S1). Close to the extinction/ survival boundary, survival is reduced if the chance of dying at random (such as from viral infection, [29]) exceeds a threshold - the average lifespan becomes less than the average generation time. These results are again as expected and are an important test that the simulation leads to expected biological behavior. Similarly, without carnivory, numbers may keep increasing in the presence of a large food supply(see Supplementary Figure S2), or overshoot and then stabilize when there is little food supplied (Supplementary Figure S3).

With borderline settings of the parameters that led to extinction, only primary producers occur; the resulting population is too sparse to support predators. Similarly, predation does not occur if it is an unattractive strategy compared to primary production. For example, the energy gain from a single predation event has to be significantly larger per iteration than from primary production; primary producers are able to spend a greater percentage of their time feeding compared with those searching for prey. Lowering either the sensory range of unicells, or the amount of energy they are able to be store, also makes predation unviable. All these results are expected and again show realistic outputs from the simulations.

Under some conditions the predator-prey cycles in Figure 1 lead to extinction of small unicells (prey), either because they are all eaten [29] or because of predation become susceptible to catastrophic events [30] before the large unicells (phagocytotes) die from starvation. We call this an arms race [31] and it occurs when parameter settings allow predators to sustain themselves by primary production after their prey has run out, thus preventing regeneration of small primary producers. In figure 1b an arms race occurs at the start of the simulation, but terminates as soon as the predators reach a size where they cannot sustain themselves by primary production. This arms race is not however a fundamental issue because it can be avoided by increasing the size of the simulation space, while keeping food density constant. However, in practice, the computational costs become excessive. The prime parameter that holds arms races in check is the random death factor (which could, for example, result from viruses that are not modeled separately). Because large unicells have a long generation time, they are more likely to die before replication (from random causes, such as infection) than small unicells. This suppresses large unicell populations, allowing small unicells to re-establish.

Finally, we see evidence for frequency dependent selection/game theory; the optimal parameter values depend on other members of the population. For example, there is a niche available for slightly larger primary producers around sizes 20–23 (Figure 2). These are larger than the optimal size for primary producers, but they have the advantage of not being engulfed by the main group of larger cells (sizes 24–27). Although they can be eaten by the very largest unicells, such unicells are extremely rare under the settings of Figure 2. The very largest unicells are virtually ‘pure’ predators, giving a three layer system of; primary producers, omnivores (opportunistic predators that are both primary producers and predators), and finally the specialist predators. Overall, all aspects of the results are realistic biologically, and mean that fundamental aspects of life history evolution are derivable from a small number of basic principles.

## Discussion

Our primary conclusion is that specialization into predators and primary producers (whatever their energy source) is expected to arise in simple biological systems. Given the wide range of initial conditions and parameter settings that lead to predation, our results are consistent with the expectation that the ability to gain energy via engulfment of other unicells evolved early during evolution. Thus from first principles, it is unlikely that there ever was an extended period (∼1–3 billion years) when there were no predators that lived by engulfing smaller cells; that is, there was no ‘Garden of Eden’. This is of course not ‘proof’ that predation existed very early in evolution; rather we see the results as supporting the expectation that ecological specialization would occur, given these fundamental principles. From our results here we cannot even exclude that phagocytotic predators existed even before DNA was the primary coding macromolecules[37].

Our simulations were kept simple in that there was little opportunity for evolving specializations that would either enhance defenses against predators, and/or increase the ability of predators to improve their detection and capture of prey. Such adaptations would arise secondarily and would reinforce that the early niche of a lineage (the evolutionary-stable niche-discontinuity concept [ESND] from Poole et al. [32]). Such potential reinforcement of lineages only strengthens the conclusion that differentiation of ecological roles is likely to be easier at early stages of evolution, before there are high levels of cell specialization. From evolutionary principles, we would not expect small primary producers to develop complex defenses against predation - until predation actually existed.

Although this study was designed specifically for the question of the origin of phagocytotic predators, it is interesting that the results have implication for other questions, including a simple version of Cope's Rule [33], [34]. This describes a tendency for some groups to become larger through time, which we see several times on Figure 1 with runaway natural selection for large size. The very large cells eventually go extinct, to be replaced by a new series of cells that follow the similar increase in size; again eventually going extinct.

The interesting feature here is that the very large members of a biota do not appear to be the progenitors for successor groups of large plants or animals. Throughout evolution, large trees have occurred in Bennettitales, Selaginellales, conifers, various dicotyledonous families, etc. But new large-bodied plants arise from smaller ones, not from the giant species of the past. Similarly, with various dinosaur groups and then large mammals, each has arisen from smaller-sized groups. Again, examining Figure 1 shows a similar pattern for small size; there appear to be a succession of groups of sizes leading to very small cells. However, additional work is required to see whether there is any reversal back to larger sizes. The important point in the present context is that relevant biological phenomena are observed additional to those for which the study was designed. Many basic biological principles appear to arise from simple basic physical principles.

Returning to the predators, we do not specify here whether such early predators were prokaryotes, or an early lineage of eukaryotes/protoeukaryotes (which we call karyotes, [35]) because we wish to focus on the general principles, not details of cellular organization. (We use ‘prokaryote’ for any non-eukaryotic cell, without phylogenetic implication). Bacteria and archaea have successfully adapted to a very wide range of energy sources [36] but are not known to have a role as predators in the sense of engulfing smaller cells by phagocytosis, that is the sense in which we use predation here. If there was an early predatory prokaryote group they must have gone extinct with the rise of eukaryotes.

However, it is not the place here to decide between these two alternatives. Although we favor the early predators being karyotes, and call it the ABC theory (Archaea, Bacteria and Carnivores), it is sufficient at present to establish that basic ecological principles would have been as relevant in early evolution as in the present. Our results are certainly consistent with some recent models that put eukaryotes very early [37], [38]. If the early predators (Figs 1 and 2) did eventually become the eukaryote lineage then it would mean that some features of the eukaryote cell, such as the cascade of RNA molecules processing other RNA molecules [35], [39], [40], would be very old and would help illustrate the later stages of evolution from the RNA-world to modern biochemistry. But from the present results we certainly cannot exclude a long-lost group of bacteria or archaea that were eventually supplanted by eukaryotes.

### Conclusions

Our primary conclusion, given the wide range of initial conditions and parameter settings that lead to predation, is that differentiation into predators and primary producers is likely to arise early in evolution. From first principles, it is unlikely that there ever was an extended period (∼1–3 billion years) when there were no phagocytotic predators that lived by engulfing smaller cells; that is, there was no ‘Garden of Eden’. Our results are a sharp reminder not to ignore fundamental physical and ecological principles in evolution. We know that, on an evolutionary timescale in the modern world, there are transitions between carnivory and herbivory (giant pandas, for example) and the reverse, there are transitions from photosynthetic organisms to heterotrophic parasites (apicomplexans, such as Plasmodium), and so on. For prokaryotes the ability to use all known classes of available chemical energy sources can be called the ‘law of prokaryote infallibility’ [36]. Certainly, the more specialized a lineage becomes, the harder it may be to change its basic life history parameters, but this only reinforces the conclusion that changes were likely easier earlier in evolution. That a very early population could differentiate into primary producers and predators will not be a surprise to ecologists; though it may be to molecular biologists. It is important in molecular evolution that our theories are consistent with basic physical, thermodynamic and ecological knowledge. We do not expect that there was any extended period where normal biological principles did not apply; a time with no predators that engulfed smaller cells.

## Materials and Methods

**Figure 3:**
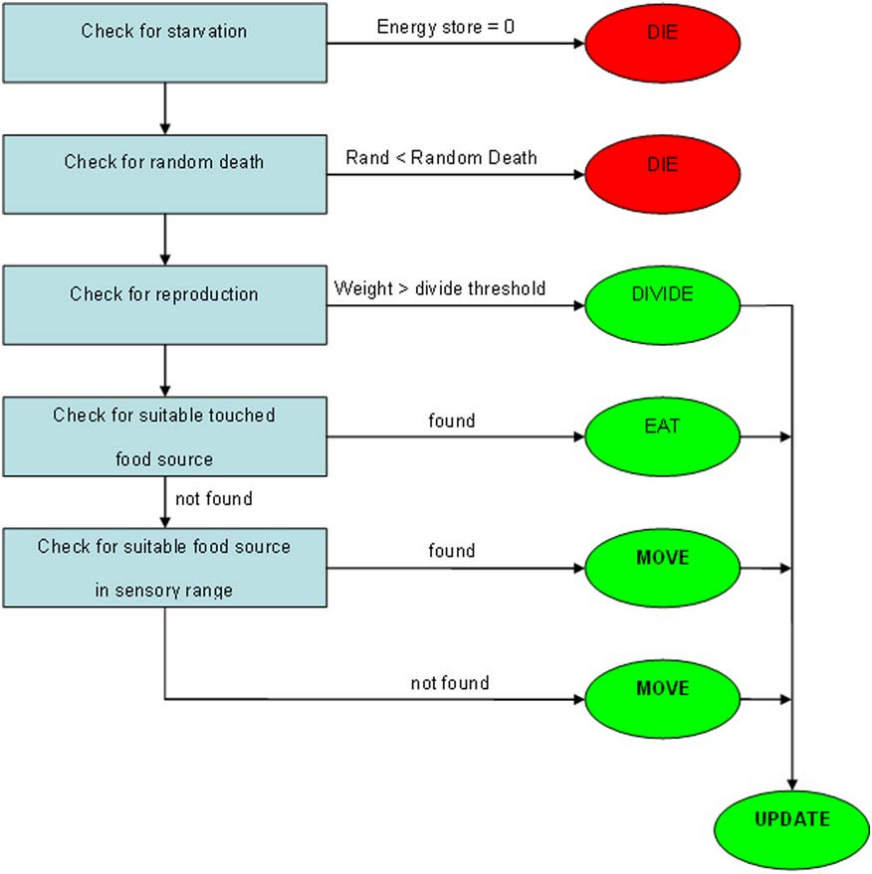
In each turn of the simulation all unicells are treated in a fixed order. If the unicell starves or dies due to the chance of random death it is removed from the simulation (though this could be modeled by saprophytes). If its energy store is sufficient then the unicell divides, creating two new unicells. These child unicells are usually half the size of their parent but there is a small chance of uneven division. New unicells cannot take any action in the first turn they are created and a unicell that divides can take no further action that turn. If a unicell doesn't have sufficient energy to divide it looks for food. First it considers what the best source of food is within its physical radius, if there is no suitable source then it will move towards the best source in its sensory radius.

We simulate the ecology of simple unicells at an undefined evolutionary stage who are able to interact only with their local environment. All unicells use the same algorithms for all their functions, and inherit the property of size from their parents. The simulation is turn-based, allowing each unicell to perform one of the following actions each iteration: feeding, division, or movement (figure 3). The simulation space, which is a two-dimensional continuous grid upon which three-dimensional cells live, contains food from which unicells can derive both energy and the basic chemicals required for growth. The food is introduced into the simulation at a constant rate and a portion of the food decays every turn. Unicells can feed on this food but also on other unicells (provided these other unicells are small enough to be engulfed).

Feeding results in energy gain. Unicells select the best energy source within the space they occupy. If this energy source is food, the amount that can be eaten is dependent on the surface area of the unicell, which is assumed to be the second power of the unicell's size. If this energy source is another (smaller) unicell then it is entirely engulfed. If no suitable energy source is available unicells will perceive whether an energy source is available within their sensory radius and move towards it, which costs energy. If no suitable energy source is perceived then unicells (weebeasties) will move in a random direction.

A limited amount of the energy obtained by feeding can be stored by the unicell. If more energy is obtained than can be stored the excess is converted into biomass. In each turn energy is lost from the store due to metabolism. When a unicell has grown enough, which on average means that it has grown to twice its original size (although variation occurs), it divides, producing two offspring of half the parent's weight (with a chance of producing an unequal division). The offspring therefore have approximately the same size as their parent started with; meaning size is an inherited property.

There are three causes of death: being eaten, starving due to their energy store reaching zero, and dying from natural causes (a random chance every turn, which includes viral infection). The corpse of the unicell is removed from the simulation in case of the latter two causes of death, but could be modeled by saprophytes. The program is written in C++, and is available from the first author (Silvester.deNooijer@wur.nl). Table 1 reports some effects of changing single parameters while keeping the others standard. In the following section a description of the parameters is given.

**Table 1:** Some standard values for parameters, and their effects.

Parameter description	Standard parameter value	Extinction	Only primary producers	Primary producers & specialized carnivores	Arms race out of control
Food increase per surface unit per turn	0.125	0–0.02	0.02–0.05	>0.05	—
Fraction of food decayed per turn	0.0005	>0.06	<0.06, >0.03	<0.03	—
Prey assembly efficiency	1.0	—	0–0.1	0.1–1.0	—
Food assembly efficiency	0.02	0–0.012	0.012–0.015	0.015–0.025	>0.03
Diameter ratio required for carnivorism	2.0	—	>3.5	<3.5	—
Multiplier to metabolism in case of movement	2.0	—	>20.0	<20.0	—
Food store size	50.0	0	>0–5	>5	—
Weight dependent metabolism factor	0.0001	>0.0085	—	<0.0085	—
Random death chance (per turn)	0.001	>0.002	<0.002, >0.0016	<0.0016, >0.0007	<0.0007

Note: The standard values give rise to a stable cohabitation of primary producers and carnivores. The last four columns show what value ranges for each parameter lead to the four distinctive behaviors (given that the other parameters are kept at standard values). The last column only shows those cases in which arms races lead to a unicell population too small in numbers to support carnivores.

### Food density

In general, scaling the dimensions of the simulation space up or down does not affect the ecology as long as food density is not changed. The numbers of unicells sustained then scales linearly with the surface size of the space. Large simulations are able to sustain larger carnivores than smaller simulations and in very small simulations with low food density there may not be enough space to support a viable population of unicells.

Lowering food density benefits primary producers and inhibits carnivores because primary producers will be more spread out spatially, and therefore carnivores have more trouble finding their prey. As a result, smaller unicells will be able to survive whereas large carnivores are unable to find sufficient prey, lowering the average size and the prey/predator ratio. Increasing food density has inverse effects, but if the food density is further increased in the standard situation, the number of primary producers increases only slightly. In this situation the number of primary producers is limited by predation and the excess food will decay instead of being eaten.

The amount of food available to unicells at any given moment can be adjusted in two ways: by increasing the amount of food introduced per turn or by decreasing the amount of food decay. The effect of these is in general the same; however, increasing the decay rate has less effect because unicells will always be able to eat some food before it decays, whereas they never can eat food that has not been introduced.

### Detection range

Even with no perception range unicells will still be able to interact with anything they touch. Randomly moving around until reaching a new food source is a viable strategy for primary producers due to the relative abundance of food in the standard situation. Carnivores need to be able to sense their prey from a distance, their chance of stumbling into prey is too low. Therefore in simulations with low or no detection range, a stable primary producer population will be observed. In the standard situation the detection range of a unicell is five times its radius, but changing the detection range to only 2 times the unicell radius has no effect on the global ecology. However, in simulations where food and therefore primary producer density is lower, a larger detection range is required for carnivore viability.

### Carnivorism efficiency

The actual value of carnivorism efficiency does not seem to be a major factor in the overall behavior of the unicell ecology. Rather, it is the ratio between primary production efficiency and carnivorism activity that matters. A high ratio means carnivorism leads to a higher energy gain per turn, and vice versa. If the ratio becomes low only primary producers and opportunistic carnivores will be found. If carnivorism results in less gain than primary production, no carnivorism will occur anymore.

### Engulfment ratio

If the diameter ratio difference required for successful carnivorism is too high, no carnivorism will occur anymore because the gains from carnivorism will become too low. However, slight changes to the engulfment ratio within the range that allows carnivorism will have a huge effect on the ecology, because carnivore energy gain scales with the third power of the prey/predator size ratio. Lower engulfment ratios will allow smaller carnivores to exist. Engulfment ratios of less than 1 will cause the carnivores to be the smallest unicells, but these values are not biologically realistic (the unicells become parasites, not predators). In general any engulfment ratio between 1.5 and 2.5 will result in omnivores and specialist carnivores.

### Carnivorism success threshold

This parameter influences the decision algorithms of the unicells by defining when a unicell is successful at carnivorism. A unicell which has derived a bigger proportion of its energy by carnivorism than the ‘carnivorism success threshold’ will not feed through primary production if there is no prey near it. Instead, it will move around randomly until it finds prey, or until it runs low on energy. The actual setting of this parameter has little effect on the ecology in the standard situation: only values above 0.8 or below 0.1 lead to reduced carnivore numbers.

### Movement cost

The metabolic cost of movement does not seem to have much effect at all on the system. Only when it becomes very high (in the order of magnitude of the size of the unicell's energy store) will it affect the viability of carnivores. Primary producers are not affected unless the value is bigger than their energy store size, in which case all unicells that move, die.

### Storage size

Storage size is defined as a certain number of metabolism turns for each unicell. In the standard situation, primary producers do not need any storage space at all. They can live and reproduce from the energy they obtain every turn. They will die as soon as all the food in their vicinity has been eaten, but by then they will have divided multiple times and because division moves the offspring unicells away from their division site, some offspring will have ended up in new food-rich areas. Nevertheless, primary producer viability increases strongly if they have a food store which is able to store just a couple of iterations worth of energy. In simulations in which food is much sparser and primary producers need to move, a food store is necessary to prevent extinction.

Carnivores cannot survive without a minimal storage space of 5, and their efficiency increases with larger storage sizes. In order to have a maximum amount of carnivores, the storage space has to be able to contain the energy for at least 50 turns of metabolism. Higher values had no detectable effect.

### Size mutation

This parameter determines what proportion of divisions will be unequal. This introduces unicells into the simulation with a wider size range than possible through equal division, and therefore allows a population which has evolved to very large sizes to recover. The value of this parameter has been kept low because high values result in many unviable unicells.

### Base metabolism

This factor is necessary to prevent unicells from evolving towards infinitely small sizes. Without it, smaller unicell sizes lead to higher surface to volume ratios and therefore to extremely fast replication, reaching the maximum replication rate of once every turn and thereby escaping predation. Whether the escape is permanent or whether predators will catch up eventually is an open question, but the exponential growth of small primary producers presents computational problems. However, regardless of this theoretical question, evolution towards infinitely small cell sizes is not realistic. Rather than posing a minimum size, we have included a metabolism cost necessary for vital functions such as maintenance of the genetic material, which all unicells have to meet irrespective of size. To compensate this fixed cost a unicell needs a certain energy influx, which can only be achieved by unicells with a certain size. Thus base metabolism functions as a lower limit on size. For unicells beyond this size, base metabolism plays only a minor role. Any base metabolism that is larger than the metabolism factor has the desired effect because arms races will dominate as soon as infinitely small size is prevented by any significant base metabolism value. Nevertheless, in the standard situation a larger base metabolism of 0.05 is used to speed up computations by preventing large numbers of small unicells.

### Metabolism factor

The metabolism factor is related to the above Base metabolism factor, and is weight-dependent. If set too high, extinction occurs because the unicells cannot consume enough food to compensate their metabolism. On the low end this factor has little effect on the system. Even if set to 0 the global ecology doesn't change because random death will eventually kill all unicells. Related are the ‘food assembly efficiency’ and ‘prey assembly efficiency’ (Tables 1 and 2). The former is the proportion of biomass consumed that is actually converted into energy for primary production and the latter the equivalent for and predation (where it it determines how much energy is lost in predator/prey interactions).

**Table 2:** The parameter values for figures 1 and 2.

Parameter values	Standard situation	Figure 1	Figure 2
Dimensions	1000×1000	10000×10000	2000×2000
Initial food per surface unit per turn	0.0125	0.005	0.0125
Food increase per surface unit per turn	0.000625	0.000025	0.000625
Fraction of food decayed per turn	0.0005	0.00025	0.0005
Initial unicell number	1000	1000	1000
Initial unicell size	15	15	Various distributions (see figure and figure caption)
Food store size	50	50	50
Initial food store contents	5	5	20
Food assembly efficiency	0.02	0.02	0.02
Prey assembly efficiency	1.0	1.0	1.0
Diameter ratio required for carnivorism	2.0	2.0	1.9
Base metabolism	0.05	0.05	0.05
Weight dependent metabolism factor	0.0001	0.0001	0.0001
Carnivorism success factor	0.5	0.5	0.5
Random death chance (per turn)	0.001	0.001	0.001
Sensory range factor	5	5	5
Multiplier to metabolism in case of movement	2	2	2
Unequal division chance	0.05	0.05	0.05
Degree of inequality parameter	2	2	2

### “Random death”

There are several biological interpretations of ‘random death’. Death could be accidental, but as mentioned in the main text the simplest interpretation is succumbing to infection from viral-like particles that are, for example, known to be present at high concentrations in the marine environment. Random death is the prime cause of death for large unicells, which escape predation because of their large size. While they get enough energy to prevent starvation, they grow extremely slowly if relying solely on primary production, reaching division size only after thousands of simulation steps. Without other factors, unicells of this size will accumulate in the simulation and will eventually drive all the other unicells to extinction through predation. To simulate natural death causes for unicells, a random death cause is introduced which effectively kills these accumulated unicells while having lesser effects on the smaller, faster replicating unicells much (because these smaller cells replicate multiple times before succumbing to random death). Some other combinations of factors are shown in Supplementary Information File ‘Text S1’.

## Supporting Information

Figure S1 Development of total number of unicells in time in case of no food being added (standard conditions as in Table S1, but with food addition set to 0). After 40 simulation turns, the initial food provided starts to run out and the population declines following an S-shaped curve, reaching extinction after 142 turns.
(0.66 MB TIF)

Figure S2 Development of total number of unicells without predatory carnivory. There is an exponential growth curve after an initial lag phase during which the initial unicell population evolves to smaller size (at which replication is much faster). Parameters are as in standard situation, except for unicell assimilation efficiency, which is set to 0. This ensures carnivorism is never an attractive strategy compared to primary production, and is thus never pursued. The initial amount of food and the amount of food added per generation are set 10 times higher than in the standard situation, while only 100 initial unicells are provided.
(0.66 MB TIF)

Figure S3 Development of total number of unicells with limited food supply and no carnivory. This shows that exponential growth stops after a while, ending with an overshoot and then reaching a stationary distribution with stochastic variations. Conditions are the same as in Figure 2, but the amount of food added is according to the standard situation of table S2 (it is therefore only 10% of that in figure S2).
(0.66 MB TIF)

Text S1 (0.03 MB DOC)
