# Supplementary figures and images for: The Emergence of Predators in Early Life: There was No Garden of Eden
Source: PLoS One. 2009 Jun 3;4(6):e5507. doi: 10.1371/journal.pone.0005507 (PMC2685975; doi:10.1371/journal.pone.0005507)

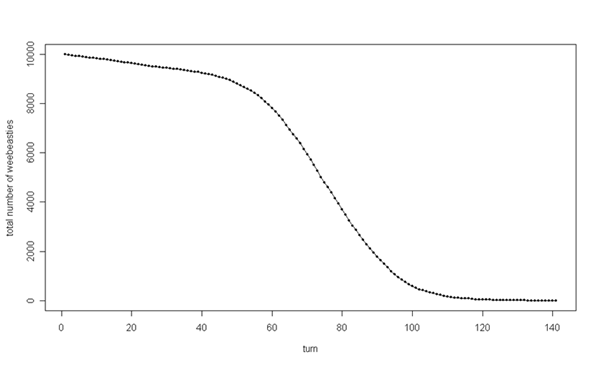

Supplement: Figure S1 — Development of total number of unicells in time in case of no food being added (standard conditions as in Table S1, but with food addition set to 0). After 40 simulation turns, the initial food provided starts to run out and the population declines following an S-shaped curve, reaching extinction after 142 turns. (0.66 MB TIF) [file pone.0005507.s001.tif]

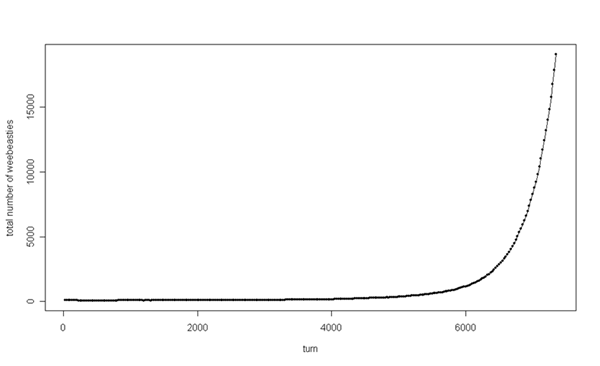

Supplement: Figure S2 — Development of total number of unicells without predatory carnivory. There is an exponential growth curve after an initial lag phase during which the initial unicell population evolves to smaller size (at which replication is much faster). Parameters are as in standard situation, except for unicell assimilation efficiency, which is set to 0. This ensures carnivorism is never an attractive strategy compared to primary production, and is thus never pursued. The initial amount of food and the amount of food added per generation are set 10 times higher than in the standard situation, while only 100 initial unicells are provided. (0.66 MB TIF) [file pone.0005507.s002.tif]

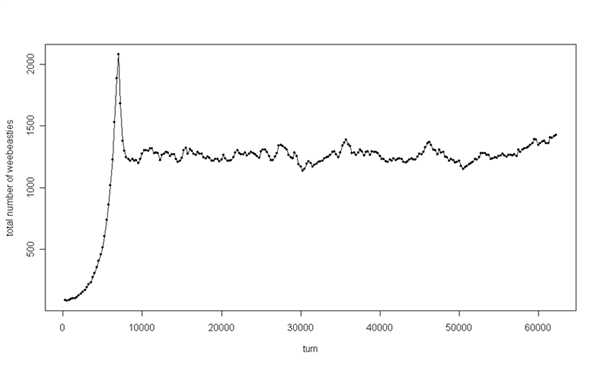

Supplement: Figure S3 — Development of total number of unicells with limited food supply and no carnivory. This shows that exponential growth stops after a while, ending with an overshoot and then reaching a stationary distribution with stochastic variations. Conditions are the same as in Figure 2, but the amount of food added is according to the standard situation of table S2 (it is therefore only 10% of that in figure S2). (0.66 MB TIF) [file pone.0005507.s003.tif]
