# Supplementary material for: The Emergence of Predators in Early Life: There was No Garden of Eden
Source: PLoS One. 2009 Jun 3;4(6):e5507. doi: 10.1371/journal.pone.0005507 (PMC2685975; doi:10.1371/journal.pone.0005507)
Supplement: Text S1 — (0.03 MB DOC) [file pone.0005507.s004.doc]

**Supporting Online Material:**

**The emergence of predators in early life: There was no Garden of Eden**

**Silvester de Nooijer^1,2,3^, Barbara R Holland^1^ and David Penny^1,4^**

**^1^ Allan Wilson Center for Molecular Ecology and Evolution, Massey University, Palmerston North, New Zealand**

**^2^ Department of Genetics, University of Wageningen, Wageningen, The Netherlands**

**^3^ address for program enquiries, Silvester.deNooijer@wur.nl**

**^4^ address for general correspondence, d.penny@massey.ac.nz.**

**Testing the simulation under well characterized conditions**

In this section we describe several tests performed on the simulation. Figure S1 shows extinction due to the absence of food. When the food runs out, most of the unicells rapidly die, though a few linger on for a while. Populations of bacteria dying in the absence of food follow similar extinction curves.

Figures S2 and S3 show exponential growth in the absence of carnivory. In figure S3, a large amount of food is provided, causing the population of unicells to expand exponentially. In figure S3, only a limited (standard) amount of food is provided each turn, and their exponential population growth ends with an overshoot, followed by convergence to a stable situation with stochastic fluctuations. These behaviors too are observed in nature in bacterial populations (Tortora, Funke and Case. 1998. pp 170-171). Thus our simulation reproduces the behavior of real-life organisms in three well-characterized circumstances, including extinction in the absence of food

Additional Reference for Supplementary Information

Tortora, G J., Funke, BR & Case, C L. 1998. Microbiology (6th ed). Menlo Park, CA: Benjamin Cummings, pp 170-171
